# Blood vessel tail artifacts suppression in optical coherence tomography angiography

**DOI:** 10.1117/1.NPh.9.2.021906

**Published:** 2022-01-24

**Authors:** Yuntao Li, Jianbo Tang

**Affiliations:** a Southern University of Science and Technology, Department of Biomedical Engineering, Shenzhen, China; b Northeastern University, Department of Bioengineering, Boston, Massachusetts, United States

**Keywords:** optical coherence tomography angiography, multiple scattering, tail artifacts suppression

## Abstract

**Significance:**

A long-standing challenge of the blood vessel tail artifacts along the axial direction prevents optical coherence tomography angiography (OCTA) for a comprehensive three-dimensional (3D) vascular mapping. Addressing the blood vessel tail artifacts issue will make OCTA to be a real 3D blood vessel structural imaging technique, which in combination with OCT-based blood flow velocity measurements will pave the way for a simpler and robust 3D imaging of the capillary transit time, one important parameter for the evaluation of micro circulation.

**Approach:**

We first described the basic principles of OCTA imaging, discussed the origin of blood vessel tail artifacts in an OCTA image, then reviewed the existing OCTA techniques for tail artifacts suppression, and at last we envisioned the potential solutions for effective OCTA tail artifacts suppression.

**Results:**

The origin of blood vessel tail artifacts is due to the multiple scattering of photons with flowing red blood cells, which elongates the light path of the dynamic signal from vessel lumen to the tail regions. High numerical aperture implementation, subtraction-based post-data processing, Hessian filtering, and high acquisition rate-based dynamic analysis methods have been proposed to address the blood vessel tail artifacts issue in OCTA.

**Conclusions:**

High acquisition rate-based dynamic analysis in combination with Hessian filtering have the potential to effectively suppress the blood vessel tail artifacts and in the meantime preserve flows in small vessels within the tail region, providing real 3D OCTA imaging of blood vessel structures.

## 1 Introduction

Blood circulation is so important that one of the vital signs of death is the stop of blood flow. Blood vessel is composed of big vessels, such as arteries and veins, and small vessels of arterials, venules, and around 40 billion capillaries that deliver most oxygen and nutrients to cells in the body.^1^ The blood vessels have diameters ranging from centimeters to the finest ones of several micrometers that are close to the size of red blood cells (RBCs). The 3D geometry of the vascular network is optimized to ensure rapid blood flow support at speed of up to meters per second in large arteries as well as slow nutrition and oxygen delivery down to <1 millimeter per second in the capillary networks.^2^ Such an amazing mechanism ensures timely and efficient feeding of the vast territory of organs in our body.

The vascular morphology of the microcirculation extends to an even higher level of complexity that the small vessels are densely and intimately connected forming a complex capillary network.^3^ Recently, the mechanism and dysfunction of small vessels have attracted wide attention as the microvasculature interacts directly with cells that the signature of pathologies may first present in the small vessels before they become detectable in the blood circulation.^4^ However, our understanding of the structural and functional information of small vessels is quite limited, mainly due to the lack of imaging techniques that can provide high spatiotemporal resolution image and functional information.

Optical coherence tomography angiography (OCTA) has emerged as a powerful tool for blood vessel imaging, especially for microvascular networks.^5^^–^^9^ In 2006, OCTA was first introduced to image the blood vessel in the posterior part of human eye,^9^ and in 2007, Wang et al.^8^ described a 3D optical angiography method (OAG) that realized 3D mapping of the cerebral vascular of an adult mouse brain. In principle, OCTA maps blood vessels using the signal dynamic contrast that signals blood vessels are changing over time due to the moving of RBCs, whereas signals from surrounding tissues are relative stable. ^10^^–^^12^ Thus, OCTA has the advantage of using an intrinsic contrast agent (i.e., RBCs) to differentiate blood vessels from surrounding tissues. With three dimensional (3D) and high-spatial resolution imaging ability of small vessels, OCTA has been widely used in a variety of studies, such as angiography of retina vessels in ophthalmology,^13^^–^^19^ cerebrovascular perfusions in neuroscience,^20^^,^^21^ cancer biology,^22^^,^^23^ and skin diseases.^24^^–^^27^

**Fig. 1:**
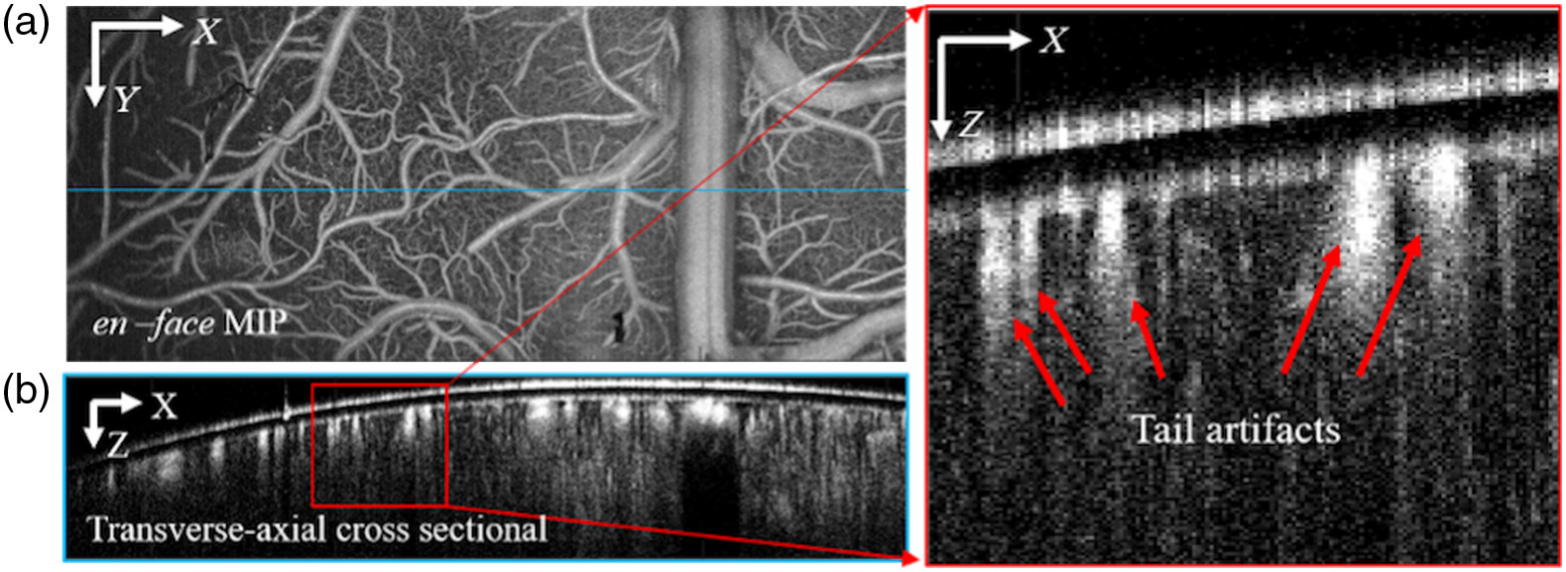
(a) OCTA en-face MIP image of cerebral blood vessels and (b) tail artifacts under big vessels challenge OCTA to realize 3D blood vessel imaging.

Despite the fast adoption in a variety of applications, OCTA is still facing several challenges, such as realizing quantitative detection of the blood flow^28^ and the tail artifacts beneath big vessels. In general, OCTA is regarded as a qualitative structural imaging technique of blood vessels. The tail artifacts beneath big vessels prevent OCTA to realize real 3D structural imaging of the blood vessel networks.^29^^–^^31^ As an example, Fig. 1(a) shows the *en face* maximum intensity projection (MIP) of cerebral blood vessels along the axial direction, which presents an elegant microvasculature image; however, the transverse-axial cross-sectional image shown in Fig. 1(b) shows notable tail artifacts beneath large vessels as indicated by the red arrows, which affects the identification of the lower boundary of large vessels and the detectability of small vessels beneath the large vessels that may be buried in the tail artifacts.

To suppress the tail artifacts in OCTA image, a variety of methods have been proposed and tested. In this paper, we will first describe the basic principles of OCTA imaging, discuss the origin of blood vessel tail artifacts in an OCTA image, then review the existing OCTA techniques for tail artifacts suppression, and at last, we envision the potential solutions for effective OCTA tail artifacts suppression.

## 2 Principle of OCTA

Doppler OCT was the first OCT-based technique to extract the velocity information from the dynamic signal of blood flow.^32^^–^^36^ However, Doppler OCT-based axial velocity detection relies on the Doppler angle that the blood flow must have a rather small angle (usually <60  deg) related to the incident light beam. Such requirement prevents Doppler OCT from detecting blood vessels that flow in nearly perpendicular direction to the light beam. In contrast to Doppler OCT that requires stable phase shift to detect axial blood flow velocity, OCTA relies on the dynamic contrast of signal variation, which can be either the phase change or intensity signal fluctuation. Thus, OCTA is able to image blood flows at any directions as long as there is signal variation.

**Fig. 2:**
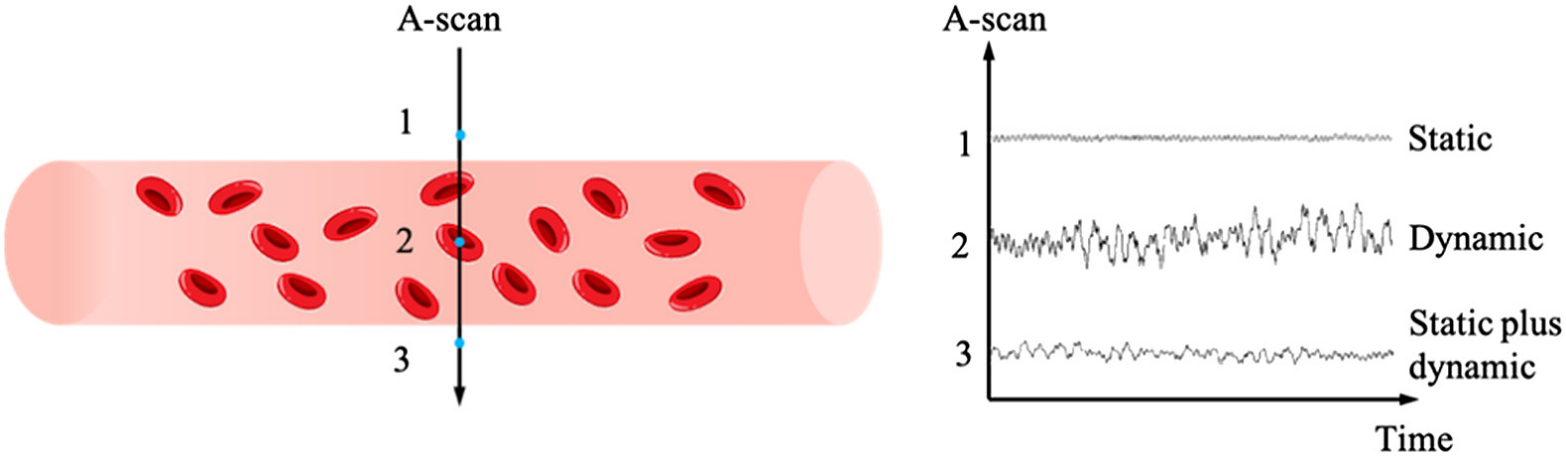
The principle of OCTA imaging. OCT signal within blood vessels (location 2) is fluctuating over time while signal from static tissue (location 1) is more stable. Location 3 is beneath the blood vessel lower boundary where mixed static and dynamic signals are presented.

Figure 2 shows the principle of OCTA imaging. Basically, the OCT signal from tissue is relatively stable compared to the signals detected within blood vessels, where the move of RBCs induces signal fluctuation. By acquiring signal from the same location at different time points the signal change within blood vessels is larger than that in the tissue, providing the dynamic contrast for OCTA imaging. Further, depending on the type of signal used for data processing, OCTA can be classified as amplitude-based,^37^^–^^39^ phase based,^40^ and complex signal-based^20^^,^^41^ techniques.

## 3 Blood Vessel Tail Artifacts in OCTA

**Fig. 3:**
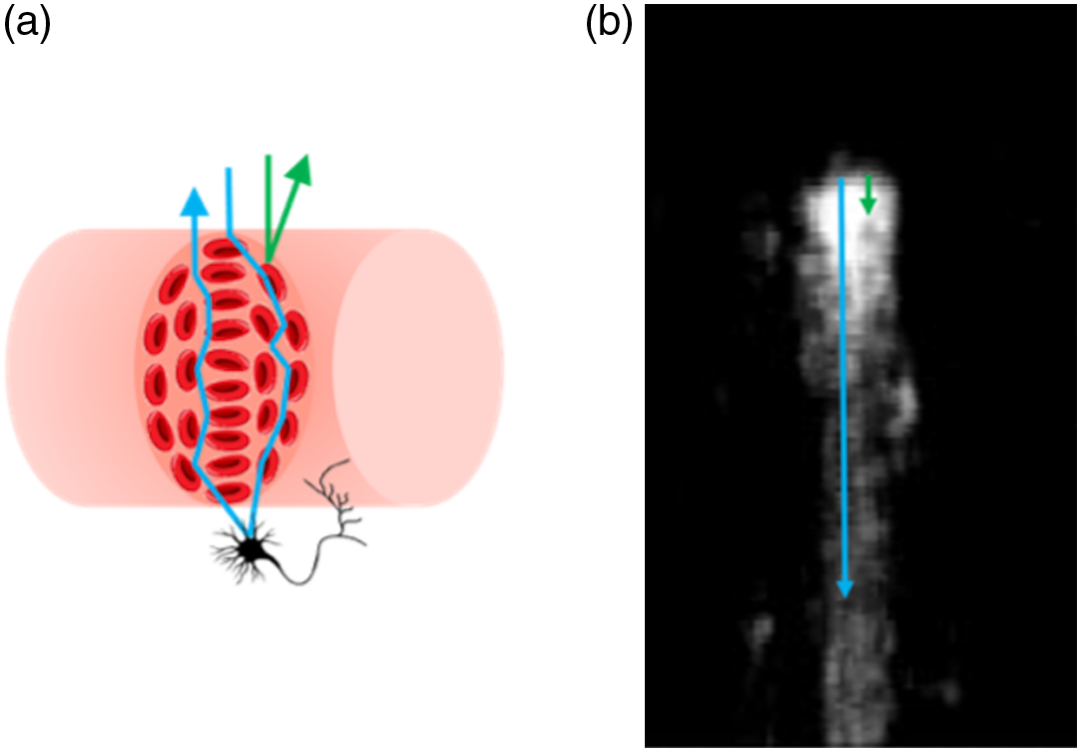
(a) Single and multiple scattering and (b) photon path length reconstruction from (a). Blue line illustrates photon experiences multiple scattering event and is collected by the objective as blood vessel signal from beneath the vessel lumen. Green line represents true blood vessel signal that photon only occurs single interaction.

OCT imaging relies on the light path match between the reference arm and the sample arm.^42^^,^^43^ The depth location of a scatterer within the sample is determined by the light path length of the photon that hits and be reflected by the scatterer.^11^ Figure 3 shows the potential interactions of an incident photon when it enters the blood vessel and in the tissue. RBCs are the main scatters within blood vessels, whose interaction with incident photons results in dynamic contrast for OCTA imaging.^44^^,^^45^ The interactions between the RBCs and photons could be classified into two types: single interaction of sole backward scattering (green line in Fig. 3) and multiple interactions of forward and backward scatterings (blue line in Fig. 3). In the former backward scattering scenario, the incident photon is reflected when it hits the RBC. Thus, the light path length of the photon has traveled represents the exact location of the RBC within the blood vessel. While for the latter scenario of mixed forward and backward scatterings, the incident photon would experience multiple scatterings before it being backward reflected by another RBC or static tissue and finally collected by the objective.^47^^–^^49^ In this case, the light path length of the photon has traveled is elongated, which may extend beyond the equivalent light path of the blood vessel lower boundary, resulting in dynamic signal beneath the vessel. As OCTA imaging relies on dynamic contrast,^46^ such dynamic signal under the blood vessels will be preserved during OCTA data processing, leading to the blood vessel tail artifacts in an OCTA image. Thus, the blood vessel tail artifacts in an OCTA image are due to the elongated light path length caused by the multiple scattering of photons that carry the dynamic information after interacted with the flowing RBCs in the vessels.

## 4 Suppress the Blood Vessel Tail Artifacts in OCTA

To realize real 3D OCTA imaging of blood vasculatures, a series of methods have been proposed to suppress the blood vessel tail artifacts in OCTA, including using high numerical aperture (NA) objectives^50^^,^^51^ to reject signals from deeper layers and many data processing algorithm-based approaches.^15^^,^^16^^,^^52^^–^^60^ Here, we review the existing methods used for blood vessel tail artifacts suppression in OCTA and the advantage and limitations of each approach.

### 4.1 Hardware-Based OCTA

**Fig. 4:**
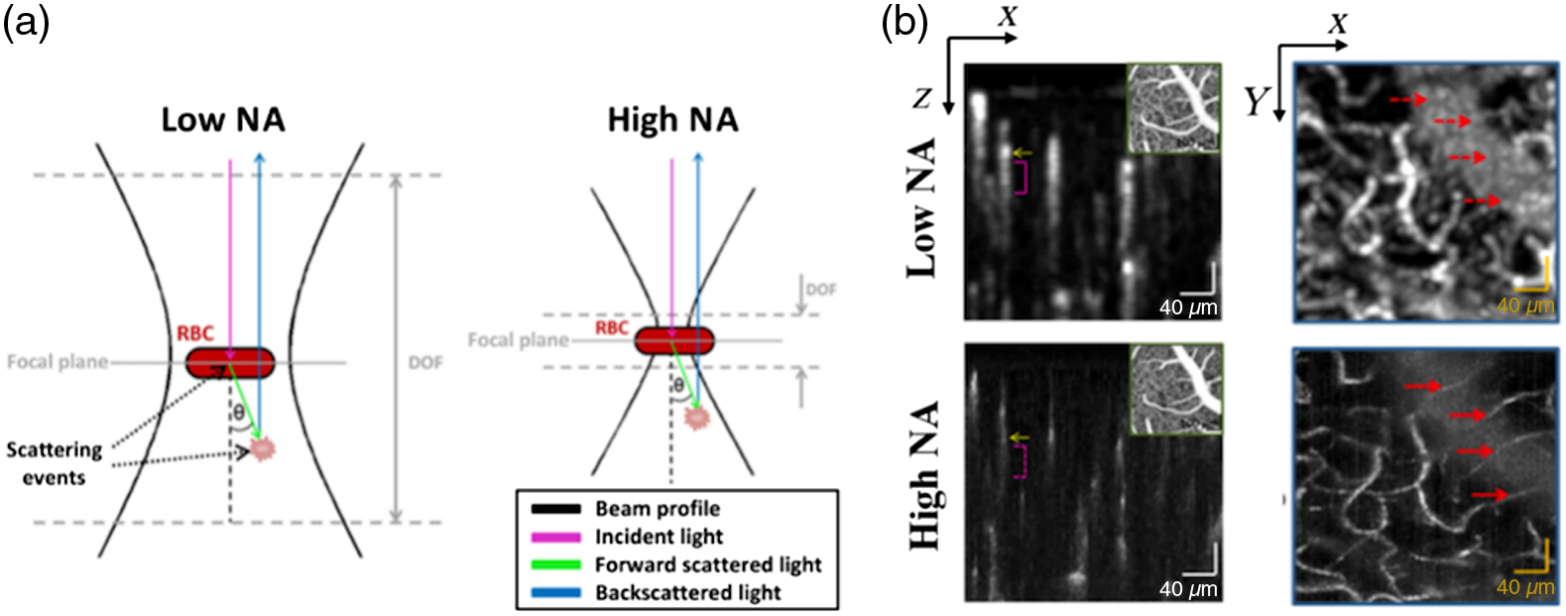
(a) Compared to the low NA objective, the high NA objective-based detection is less sensitive to multiple scattering events. (b) With high NA objective, OCM-based angiography can suppress the blood vessel tail artifacts and detect small vessels that may be buried in the tail artifacts with low NA objective-based detection. Reproduced with permission, courtesy of C. Leahy et al.

A high NA objective has a short depth of focus, and it mainly collects signal reflected from the focusing layer. Optical coherence microscopy (OCM) employs a high NA objective in the sample arm so that signal from the out-of-focus layers would be rejected. Based on this idea, Leahy et al.^50^ used a high NA objective with a depth of focus of 15.1  μm in their OCT system to perform OCTA data acquisition, giving a very thin layer of detection. Thus, photons experiencing multiple scattering and finally reflected at the out-of-focus region would not be collected by the objective, realizing the suppression of tail artifacts under the blood vessels, as shown in Fig. 4(a). Compared to the traditional low NA objective-based OCTA, this approach can largely suppress the tail artifacts [magenta bracket indicated in Fig. 4(b)] and small blood vessels underneath the large vessels were successfully detected [marked with red arrows in Fig. 4(b)]. However, the high NA objective-based OCM method needs multiple scanning at different depths to realize a 3D imaging of the vessels, resulting in extended data acquisition time. In addition, the high NA objective gives very high lateral resolution, which makes this method more sensitive to bulk motion, deteriorating the image quality.

**Fig. 5:**
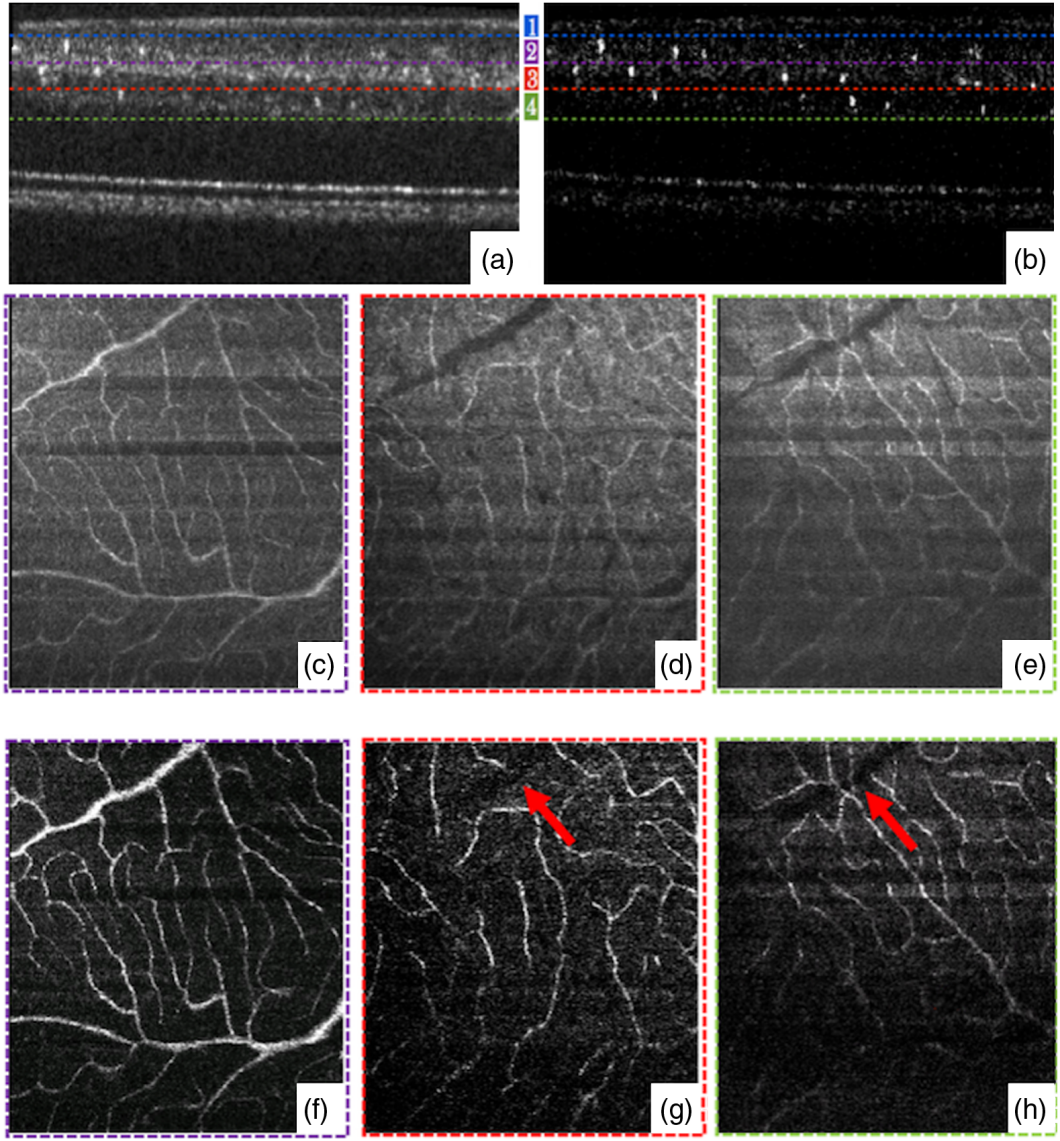
Comparison of AO-OCT and AO-OCTA. (a) and (b) B-scan images generated from AO-OCT and AO-OCTA. (c)–(h) *en face* MIP images in three different depths below the topmost layer marked in (a) and (b). Red arrows indicate tail artifacts from (f) are suppressed.

Adaptive optics (AO)-based OCT was applied to suppress the blood vessel tail artifacts as well.^51^ AO-OCTA was first described in 2012.^61^ With the advantage of high NA, Salas et al. demonstrated that AO-OCTA has the ability to suppress the tail artifacts beneath large vessels, providing a high-resolution and high signal-to-background contrast angiogram.^51^ As shown in Fig. 5, the blood vessel tail artifacts were greatly suppressed in deeper layers [Figs. 5(g) and 5(h)] with enhanced signal-to-noise ratio (SNR), suggesting the combination of AO and OCTA has the ability of reducing tail artifacts from the top layer large vessels and enhancing the contrast of blood flow in deeper layers.

### 4.2 Axial Subtraction-Based Approaches for Blood Vessel Tail Artifacts Removal

**Fig. 6:**
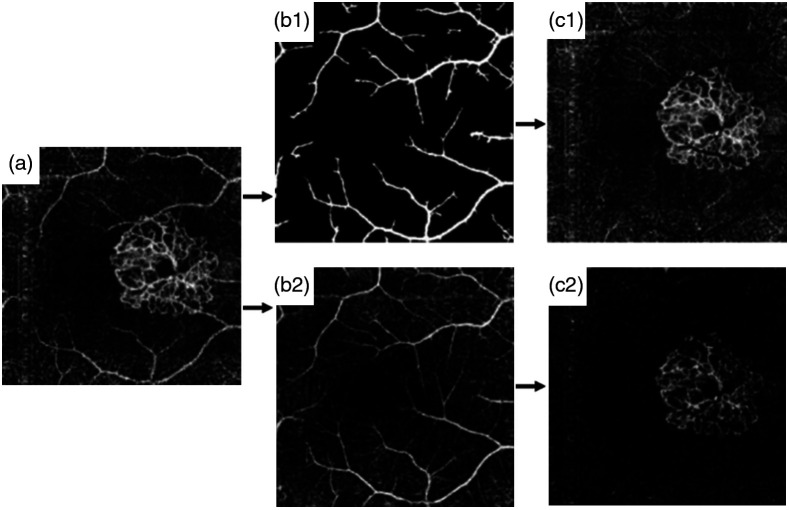
(a) Original outer retinal angiogram with CNV. (b1) and (c1) Comparison of Jia’s work and (b2) and (c2) Liu’s work. (b1) Inner retinal large vessel mask generated from binary operation. (b2) Filtered inner retina mask. (c1) Outer retina with larger vessel artifacts removed. (c2) Outer retina with artifacts removed. Reproduced with permission, courtesy of Liu et al.

As shown in Fig. 6(a), the MIP of the outer retina blood vessel angiogram is composed of blood flows in the outer retina layer and the blood flow artifacts projected from the inner retina layer. To quantify choroidal neovascularization (CNV) in age-related macular degeneration, Jia et al.^15^ introduced the slab-subtraction (SS) method to remove the projected tail flows of inner retina in the outer retina layers. In Jia et al.’s work, a binary mask of large vessels in the inner retina layer was first obtained [Fig. 6(b1)] and then subtracted from the deep layers [Fig. 6(c1)]. This method can remove the projection tail artifacts of large vessels but still be affected by the small vessel projections. Liu et al.^53^ further created a blood vessel mask of the inner retina layer [Fig. 6(b2)] and obtained a clearer blood vessel angiogram in the outer retina [Fig. 6(c2)].

**Fig. 7:**
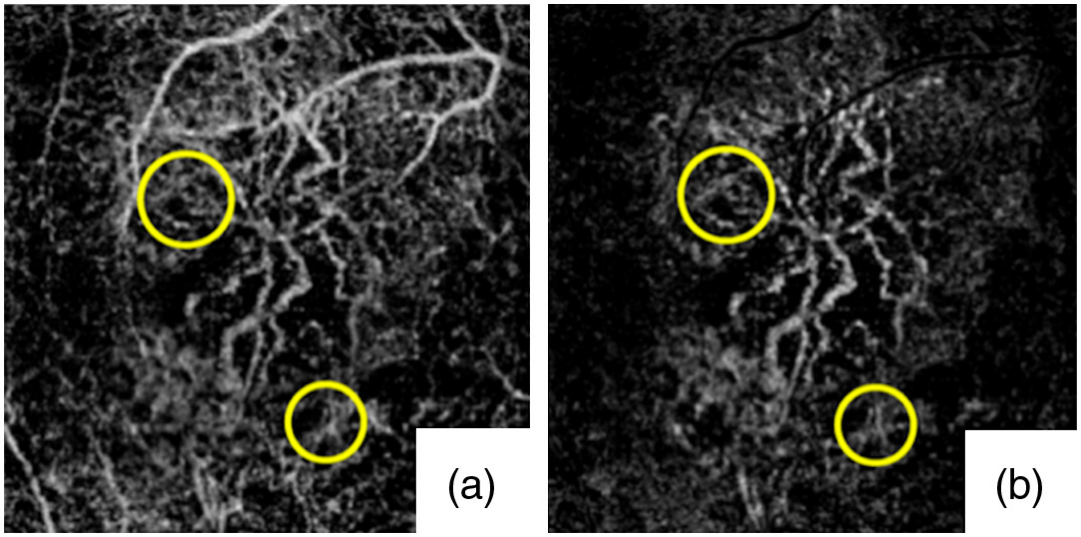
OCTA of the Type 1 CNV case indicated by the yellow circles. (a) Original OCTA of the entire outer retina. (b) OCTA of the entire outer retina after tail artifacts removed. Reproduced with permission, courtesy of Zhang et al.

The SS algorithm removes the blood vessel tail artifacts in deep layers by directly subtracting blood flow signal in the superficial layers, which, however, leads to broken flows in deep layers [Fig. 6(c)]. Zhang et al.^54^ considered the *in situ* pathological information to improve the blood flow continuity of the CNV.

$$\log[A_{T}(x,y)]=\log[A_{s}(x,y)]\cdot \{1-\text{Norm}\;\langle \log[A_{R}(x,y)]\rangle \cdot \;\;\{1-\text{norm}\langle \;\log[I_{S}(x,y)]\rangle \}\},$$ (1)

where $A_{T}(x,y)$ is the true signal of interest, $A_{s}(x,y)$ is the detected projection image from outer retinal avascular space (ORAS), $A_{R}(x,y)$ is the projection image obtained from retina, Nm⟨…⟩ represents normalization operation, and $I_{S}(x,y)$ is the structural projection image from the ORAS. Figure 7 shows that this improved SS method can remove the blood flow projection tail artifacts that may mislead diagnosis, and preserve the neovascular indicated by the yellow circles [Fig. 7(b)].

Choi et al.^57^ further proposed a mean-subtraction method using a weighted mean value of all pixels of each A-line to suppress the signal in the subluminal area of the large blood vessel. Mathematically, this mean-subtraction method is expressed as

$${\mathrm{OCTA}}_{\mathrm{DS}}(i)=\mathrm{OCTA}(i)-\omega \times \frac{1}{N}\overset{N}{\underset{k=1}{\sum }}\mathrm{OCTA}(i,k).$$ (2)

where ${\mathrm{OCTA}}_{\mathrm{DS}}(i)$ and OCTA(i) represent the i’th A-lines of the de-shadowed and original image, respectively; OCTA(i,k) is the magnitude at the k’th pixel point of the i’th A-line of the original image; k is equal to the number of all pixel points of the A-line; ω is a weighted average, which was set to 2.

**Fig. 8:**
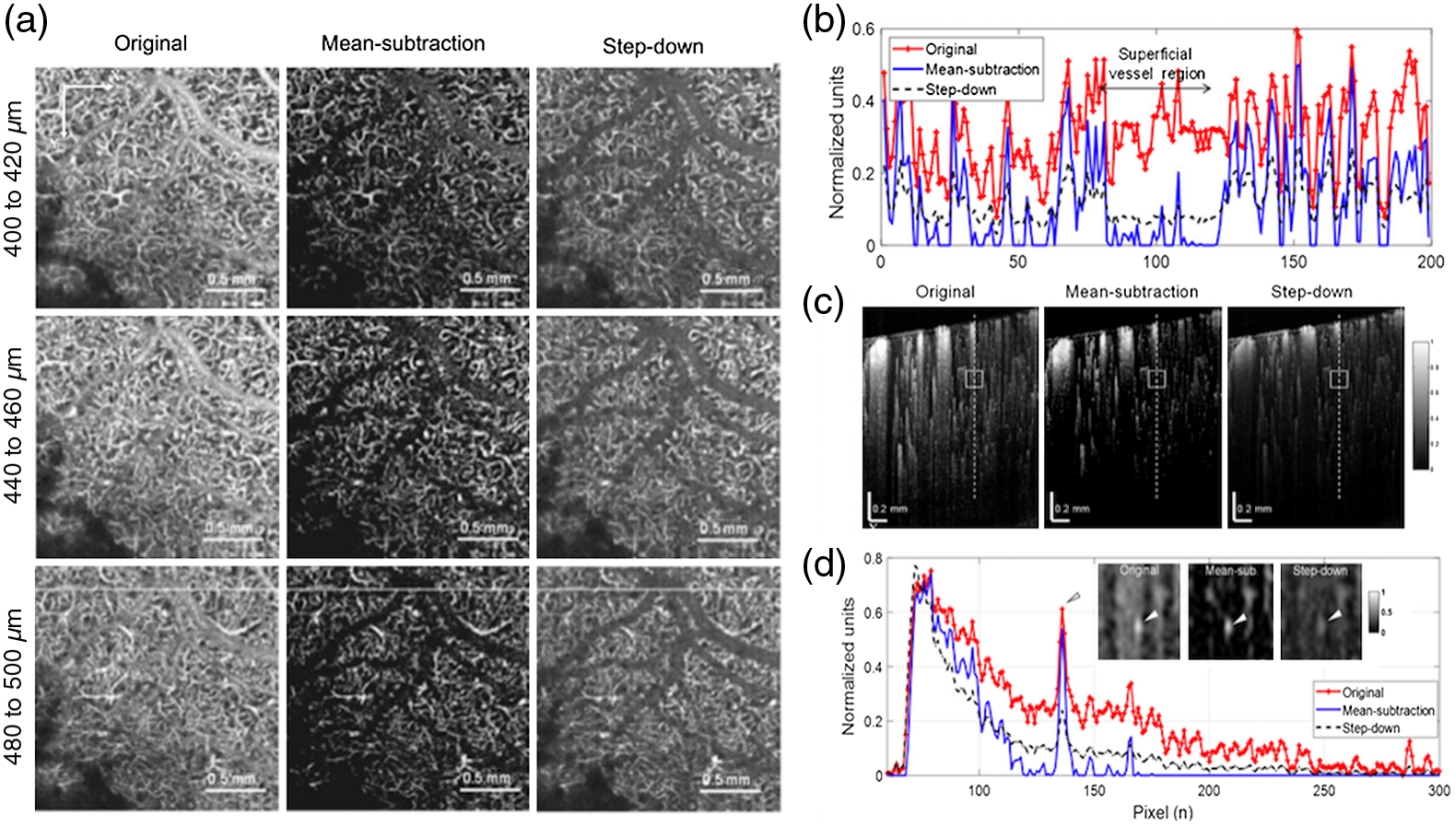
(a) Depth-specific comparison of original and artifacts-removal *en face* OCTA images used the mean-subtraction method and the step-down method. (b) Taken along horizontal white lines on the OCTA images at the bottom of (c). (c) Normalized OCTA cross-sections of mouse cortex: original (left), processed with the mean-subtraction method (middle) and the step-down method (right). (d) Depth profiles taken along the dashed lines in (c) showing largely reduced tails for both methods. Inserts in the graph are regions of interest indicated as boxes in (c), and arrows represented underlying vessel, which is brighter and easily identified with the proposed method. Reproduced with permission, courtesy of Choi et al.

Compared to the step-down exponential filtering method, which obscured small vessels underneath the large vessels,^23^ the mean-subtraction method showed higher signal-to-background ratio (Fig. 8). However, similar to the aforementioned subtraction-based methods, the mean-subtraction approach is able to remove the blood vessel tail artifacts projection in deep layers but still at the cost of broken flows. This method works fine for qualitative evaluation and manual assessment, but it’s challenge for quantitative analysis of the blood flow structure in deep layers.

### 4.3 Projection-Resolved-OCTA and Reflectance-Based PR-OCTA

**Fig. 9:**
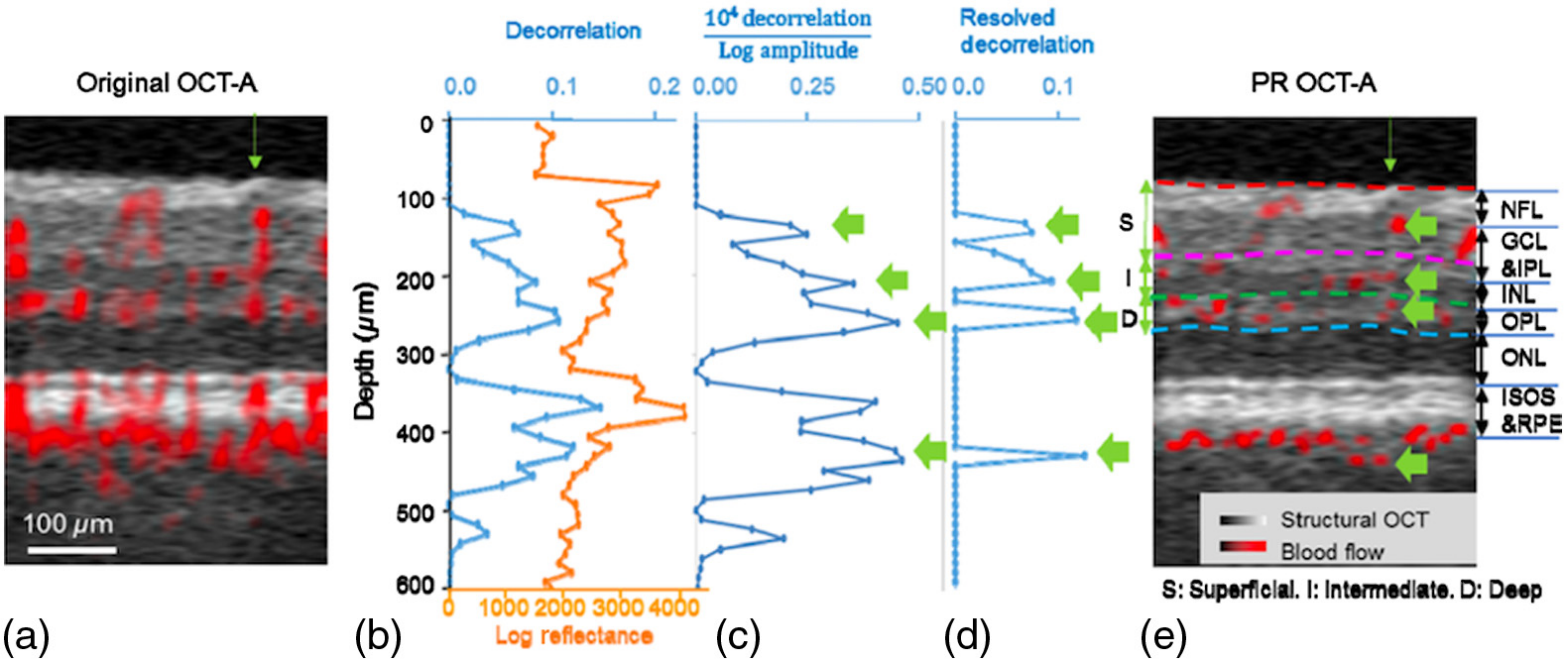
Illustration of PR-OCTA for blood vessel tail artifacts suppression. (a) Original cross-sectional image before artifacts suppression. (b) Original decorrelation and log amplitude values of the A-line pointed to by the green arrow in (a). (c) Decorrelation normalized by log amplitude according to data shown in (b). Four highest peaks (green arrows) in this curve were identified as blood vessels. (d) Decorrelations plot after cleaning up by the PR algorithm–decorrelation values besides vessels were set to zero. (e) Cross-sectional OCTA image after PR-OCTA processing. Four vessels are identified with green arrow, as shown in (c) and (d). Reproduced with permission, courtesy of Zhang et al.

**Fig. 10:**
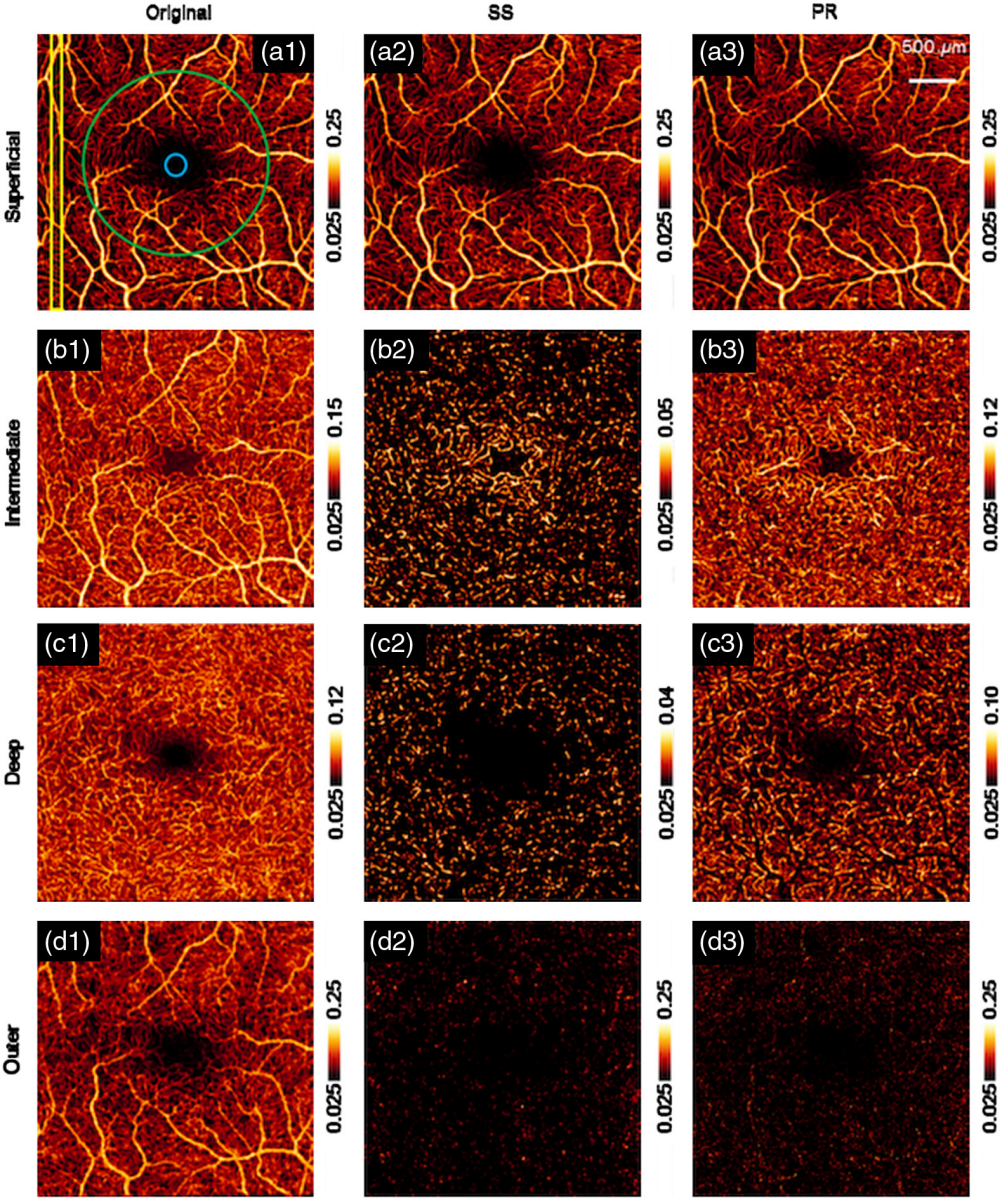
OCTA *en face* images with original, SS method and PR method in superficial vascular plexus (a1)–(a3), intermediate capillary plexus (b1)–(b3), deep capillary plexus (c1)–(c3), and outer retinal region (d1)–(d3). individually. Reproduced with permission, courtesy of Zhang et al.

To minimize the broken flow issue in axial subtraction-based method, Zhang et al.^55^ further proposed a projection-resolved (PR)-OCTA approach that finds local peaks along an OCTA A-line to subtract projection tails (Fig. 9). In principle, the tail artifacts signal is always weaker than the signal from vessel lumen. Thus, by searching for peaks from the normalized signal F on each A-line and set the tail region to 0, the PR-OCTA can preserve more flow voxels under larger vessels, which may be removed by the SS method. The PR-OCTA method can be described as Eq. (3).

$$C_{n}=\{\begin{array}{ll} D_{n}, & \text{if}\;F_{n}>(1+\alpha )\max(F_{i}),\;1\leq i\leq n-1\\ 0, & \text{otherwise} \end{array},$$ (3)

where i and n are the index of a voxel in A-line from the top beginning (n=512 in this method). C is the corrected decorrelation value after PR algorithm processed. D is the normalizing decorrelation. In this operation, α is included to account for noise and it was set to 0.1. Figure 10 compares the results obtained with the SS method and PR-OCTA.

**Fig. 11:**
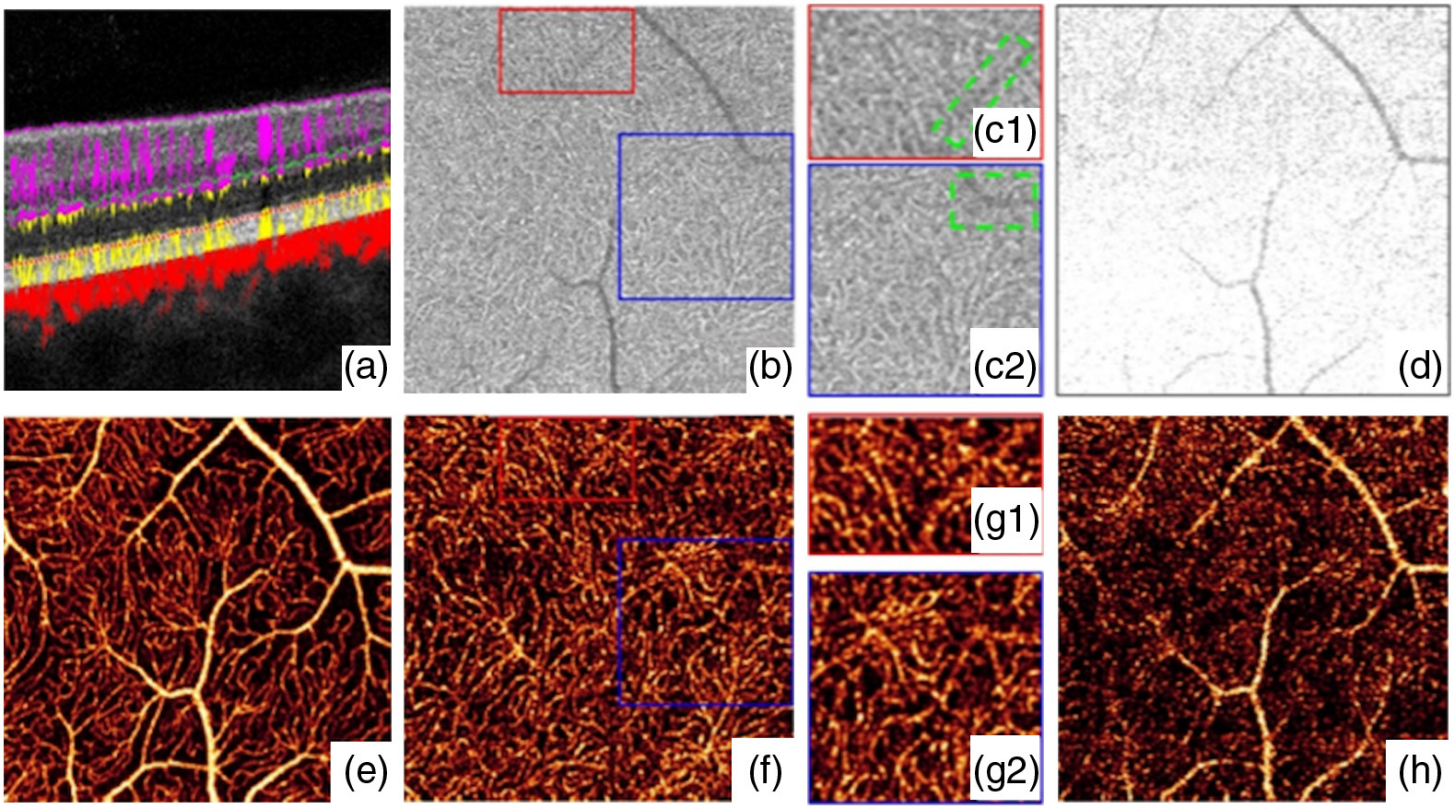
Illustration of the relationship between vessels, shadow artifacts, and the projection artifacts. (a) Cross-sectional structural OCT (gray) overlaid with OCTA (red). (b) OCT reflectance in deep plexus slab, highlighted by yellow dotted line in (a). (c1) and (c2) Enlarged regions in (b) to show the vessels shadows artifacts with low reflectance (green-dashed square) and the capillary with high reflectance. (d) OCT reflectance in outer retinal slab, highlighted by green dotted line in (a). (e) The MIP of OCTA in superficial plexus slab, horizontal white dashed line indicates the location of panel (a). (f) OCTA in deep plexus slab. (g1) and (g2) Enlarged regions in (f) to show the real capillaries interfered by projection artifacts. (h) OCTA in outer retinal slab. Reproduced with permission, courtesy of Wang et al.

**Fig. 12:**
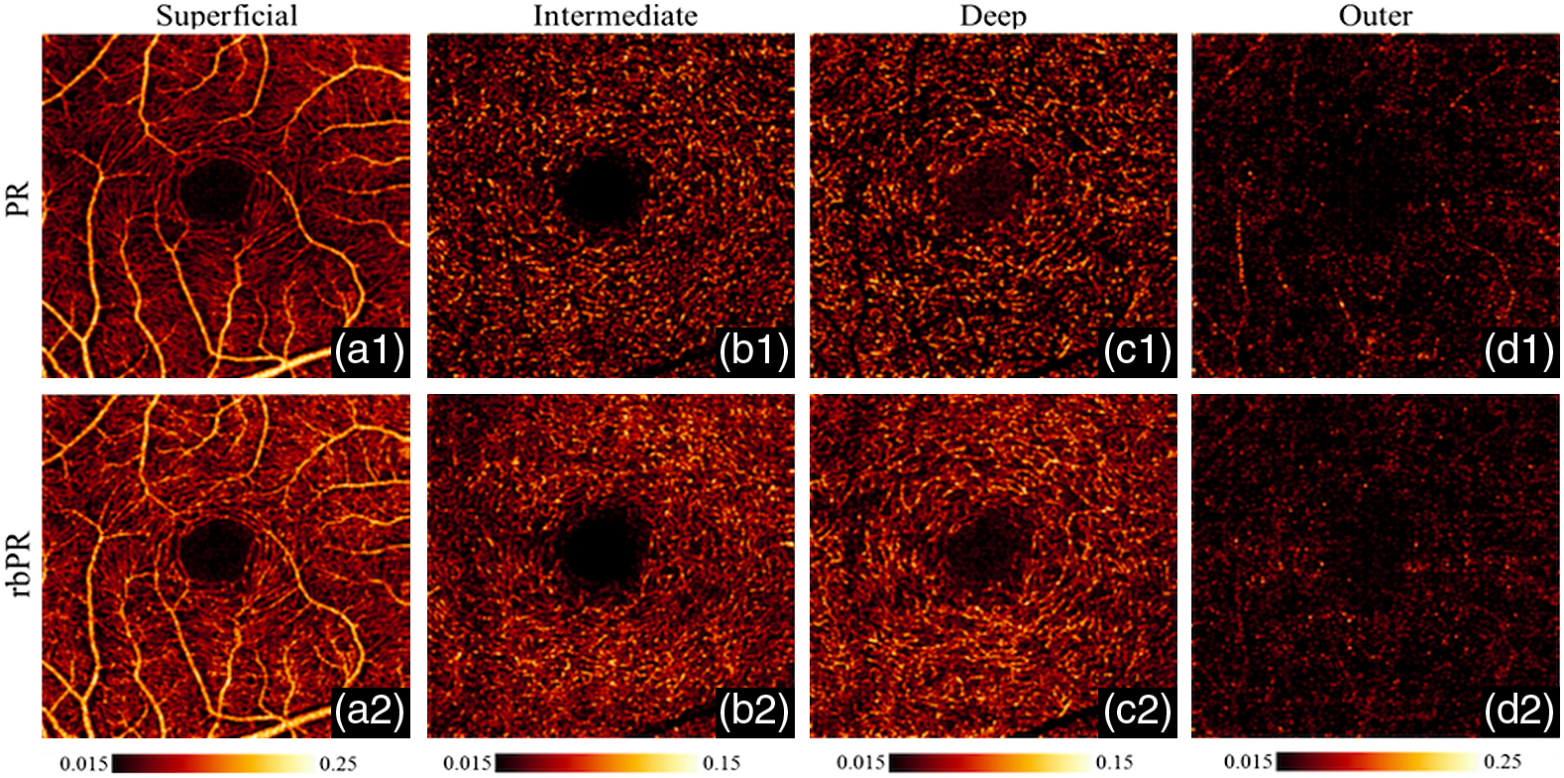
A comparison of retinal OCTA images from a normal eye processed with tail artifacts suppressed by the PR method (a1)–(d1) and the proposed rbPR method (a2)–(d2) in three distinct layers. Reproduced with permission, courtesy of Wang et al.

The PR-OCTA preserved more blood vessel structures under the superficial large vessels, but still faced the problem of discontinued flows in deep layers [Fig. 10(c3)]. Wang et al.^56^ proposed a reflectance-based PR (rbPR)-OCTA algorithm to further improve the blood flow continuity by taking the OCT structural reflectance signals into account. The rbPR-OCTA uses the information from both OCT structure image and OCTA result to more accurately determine and remove the blood vessel tail artifacts. As shown in Fig. 11(b), in the deep layers of OCT structure images, there exist shadows of superficial blood vessels that have lower reflectance compared to surrounding tissues. In contrast, in the OCTA images [Fig. 11(f)] those shadow regions are brighter than the surrounding tissue regions. Hence it is feasible to obtain the reflectance information from OCT structure image to enhance the contrast of true vessel structure and suppress tail artifacts in OCTA images. The rbPR-OCTA approach was first to segment the scan volume into sub-volumes of above and below inner/outer segments (IS/OS). The sub-volume above IS/OS was then scaled by normalized reflectance-based vessel contrast map (obtained from a K-means clustering method) to suppress tail artifacts, and the sub-volume below IS/OS was scaled by vessel probability distribution map (obtained from a fuzzy C-means method) to enhance the vasculature. Compared to PR-OCTA, the rbPR-OCTA showed a better neovascularization continuity visualization (Fig. 12). It distinguishes blood flow signals and projection artifacts by analyzing *en face* images slice by slice rather than processing each A-line. This method improves the blood flow continuity in the outer retina.

### 4.4 Shape Filtering-Based Blood Vessel Tail Artifacts Suppression

Shape filtering-based methods such as the Hessian filtering also show the ability to suppress the blood vessel tail artifacts in OCTA.^60^^,^^62^^,^^63^ To image capillary and analyze its network flux, Lee et al.^62^ first resampled the axial pixels so that the OCT image voxel has the same size in both axial and transverse directions. Then Hessian filtering was applied on the more circular-like vessels instead of the original teardrop-like vessel cross sections. However, the depth-compression method led to the loss of details of small vessels. In the same year, Yousefi et al.^63^ combined 3D-based Hessian filtering and intensity-based segmentation with a morphological segmentation technique to achieve the goals of slab segmentation and 2D angiography.^5^ This 3D-based Hessian filtering applied a 2D Hessian filtering to each depth-specific *en face* image that resampled from the 3D angiograms. This method can minimize the tail artifacts but the 2D Hessian analysis is less sensitive to the 3D structure due to the lack of 3D information.

**Fig. 13:**
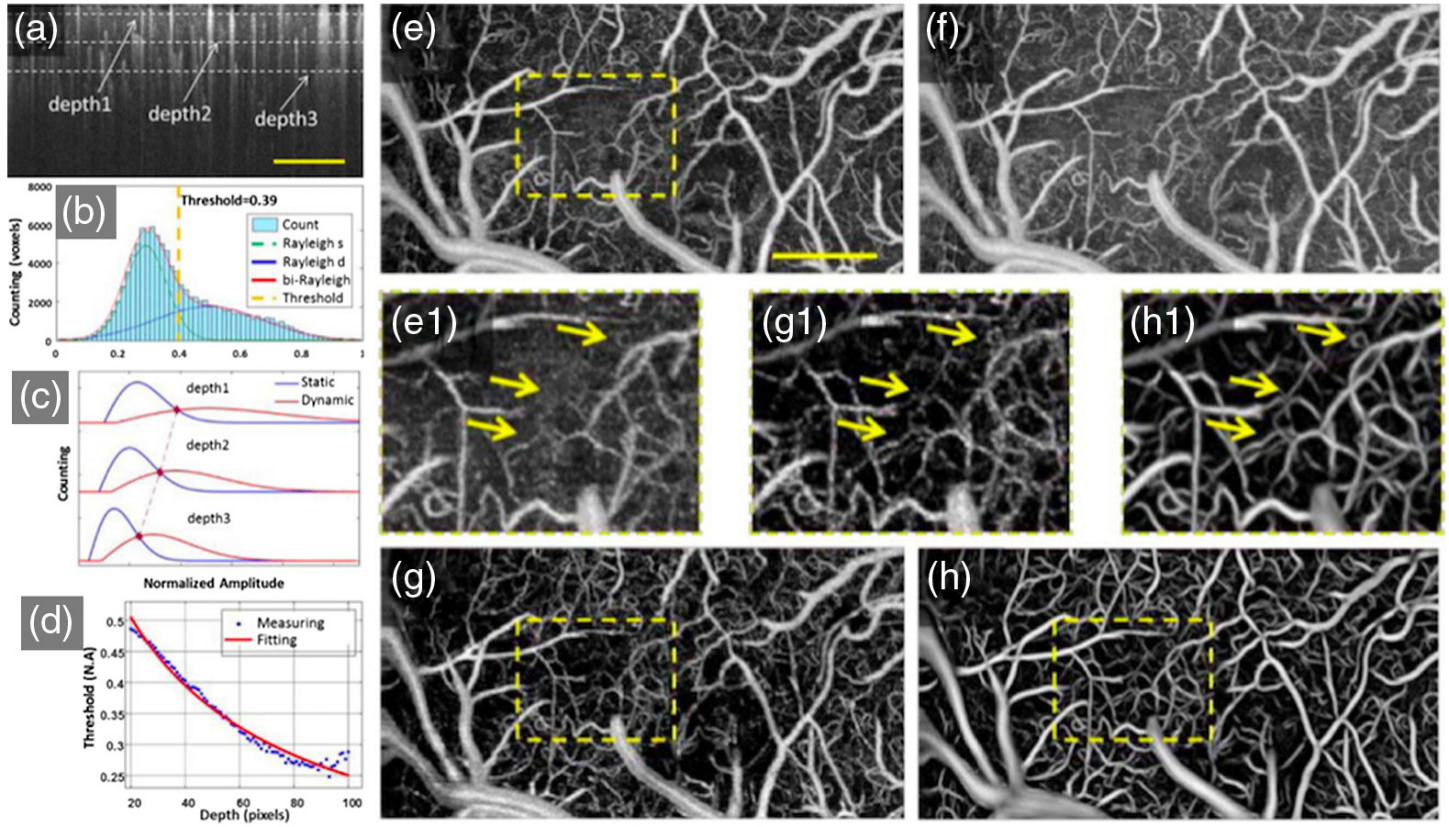
Adaptive OCTA classifier using a histogram-based motion threshold and 3D Hessian analysis-based shape filtering. (a) Representative original OCTA cross-sectional angiogram. For the convenience of analysis, three representative depth positions (depth1, depth2, and depth3, marked by the dotted lines) were selected. (b) Histogram of en-face OCTA angiogram at Depth1. The voxel populations of static tissue and dynamic flow can be identified by fitting the histogram analysis. (d) Depth dependence of the OCTA statistics and the motion threshold. MIPs of the 3D OCTA angiogram using the uniform motion threshold of (e) depth1 and (f) depth3, and (g) the depth-adaptive threshold. (h) OCTA angiogram using depth-an adaptive threshold and 3D Hessian-based shape filtering. The scale bar=400  μm. Reproduced with permission, courtesy of Li et al.

In 2017, Li et al.^60^ introduced a 3D Hessian analysis-based method for OCTA data processing. This method adaptively applied different thresholds to depth-specific slices. In each slice, OCTA signals follow Rayleigh distribution.^64^ By analyzing the number distribution of static and dynamic voxels, the motion threshold was first determined, then the 3D adaptive threshold was obtained by fitting the distribution curves [Figs. 13(a)–13(d)]. After discriminating dynamic regions, the blood vessel structure could be obtained but containing blurred vessel information in deep depth due to tail artifacts. A shape filtering based on eigenvalues and eigenvectors of the Hessian matrix was then applied to detect the tubular-like structures. The modified vessel function was defined as

$$\nu_{0}(s)=\{\begin{array}{ll} 0, & \text{if}\;\lambda_{3}>0,\\ \exp(-\frac{R_{A}^{2}}{2\alpha^{2}})\exp(-\frac{R_{B}^{2}}{2\beta^{2}})[1-\exp(-\frac{R_{c}^{2}}{2\theta^{2}})], & \text{others} \end{array},$$ (4)

where $R_{A}=|\lambda_{2}|/|\lambda_{3}|$, and $R_{B}=|\lambda_{2}|/\sqrt{\left|\lambda_{2}\lambda_{3}\right|}$. $\lambda_{k}$ is denoted as the eigenvalue with the k’th smallest magnitude of the Hessian matrix. The Frobenius matrix norm $R_{C}=\sqrt{\sum_{k\leq D}\lambda_{k}^{2}}$ is used to eliminate the effect of background noise. α, β, and θ are the corresponding thresholds and 0.5, 0.5, and 0.2, respectively. In addition, a Gaussian probe kernel was used in vertical direction to detect vessels with elongated tails and preserve vessels with short tail artifacts. Compared to the uniform motion threshold method of the 3D OCTA technique [Fig. 13(e)] and depth-threshold method without 3D Hessian filtering [Fig. 13(g)], the proposed method [Fig. 13(h)] distinguishes dynamic and static signals and preserves the details of deep vascular networks.

### 4.5 Deep Learning-Based OCTA

**Fig. 14:**
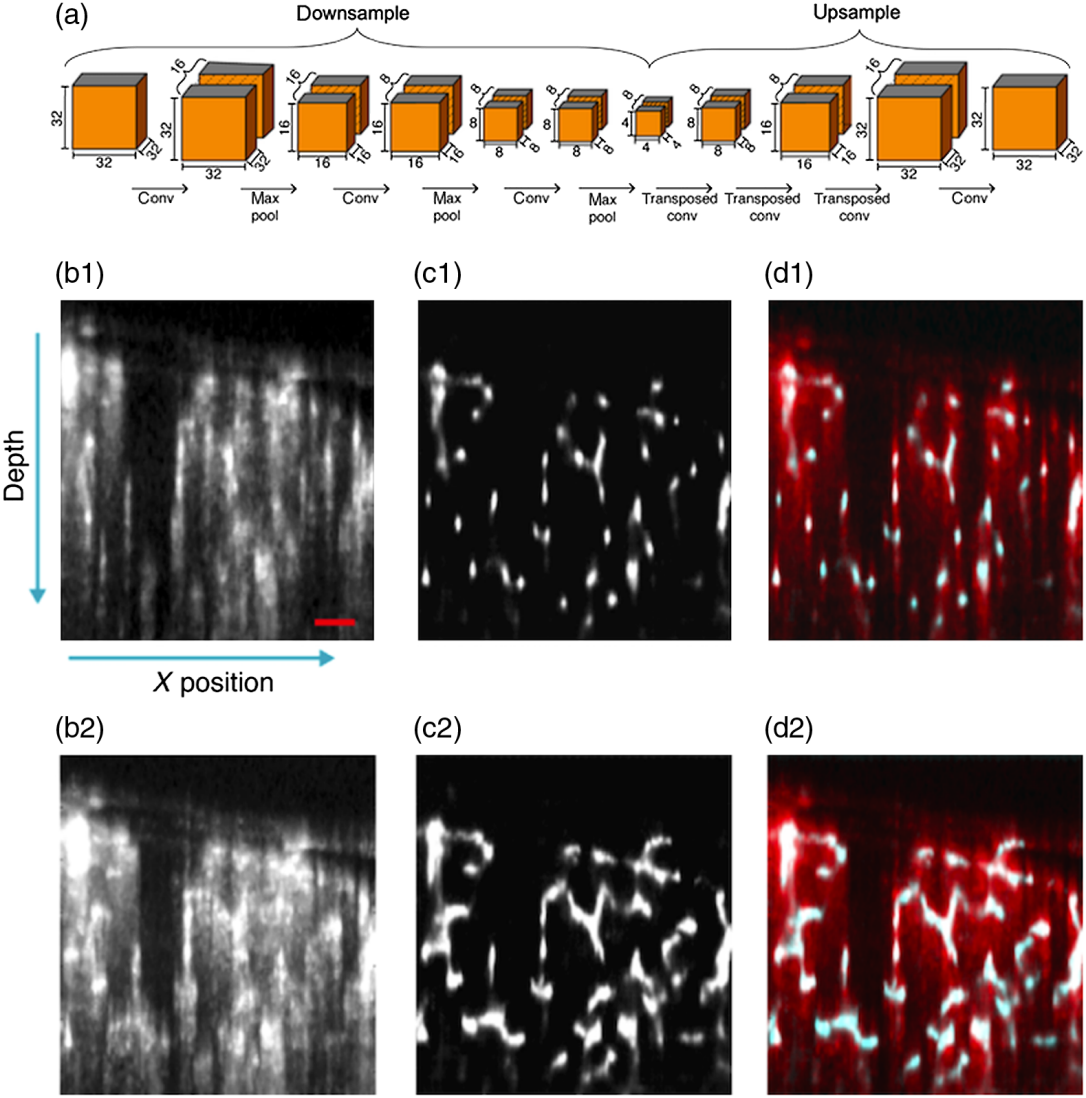
(a) Schematic description of encoder–decoder architecture in EnhVess. (b1)–(d2) Comparison of tail artifacts suppression with the original. (b) OCTA, (c) EnhVess, and overlaid result from (b) and (c). (b1)– (d1) Single B-scan images, and (b2) –(d2) MIP over 10 B-scan images. Scale bar, 0.1 mm.

Recently, Stefan et al. proposed a deep learning-based OCTA (EnhVess)^52^ to suppress the blood vessel tail artifacts and enhance the vessel connectivity, as shown in Fig. 14. The ground truth of the vessel structures was first manually labeled to train the network using a mean-squared loss function. Figure 14(a) shows how the encoder–decoder is used in EnhVess network to enhance blood vessel network while minimizing tail artifacts. Basically, a 32×32×32 3D patch of volumetric image were input to the encoder with three 3D convolutional layers followed by a max pool layer, and then the down-sampled output from encoder is decoded by three transposed convolution layers and up-sampled to a size of 32×32×32. After applying the EnhVess to a new OCTA dataset, the vessel tail artifacts are greatly suppressed, as shown in Figs. 14(b)–14(d).

This deep learning-based OCTA data processing shows the ability to suppress tail artifacts and enhance the blood flow SNR. But the main limitation of deep learning-based OCTA technique is the dependence of ground truth.^65^^,^^66^ Inaccuracy of manual segmentation and the large variation of blood vessel structures usually lead to degraded performance.

### 4.6 Normalized First-Order Field Autocorrelation Function (g1)-OCTA

**Fig. 15:**
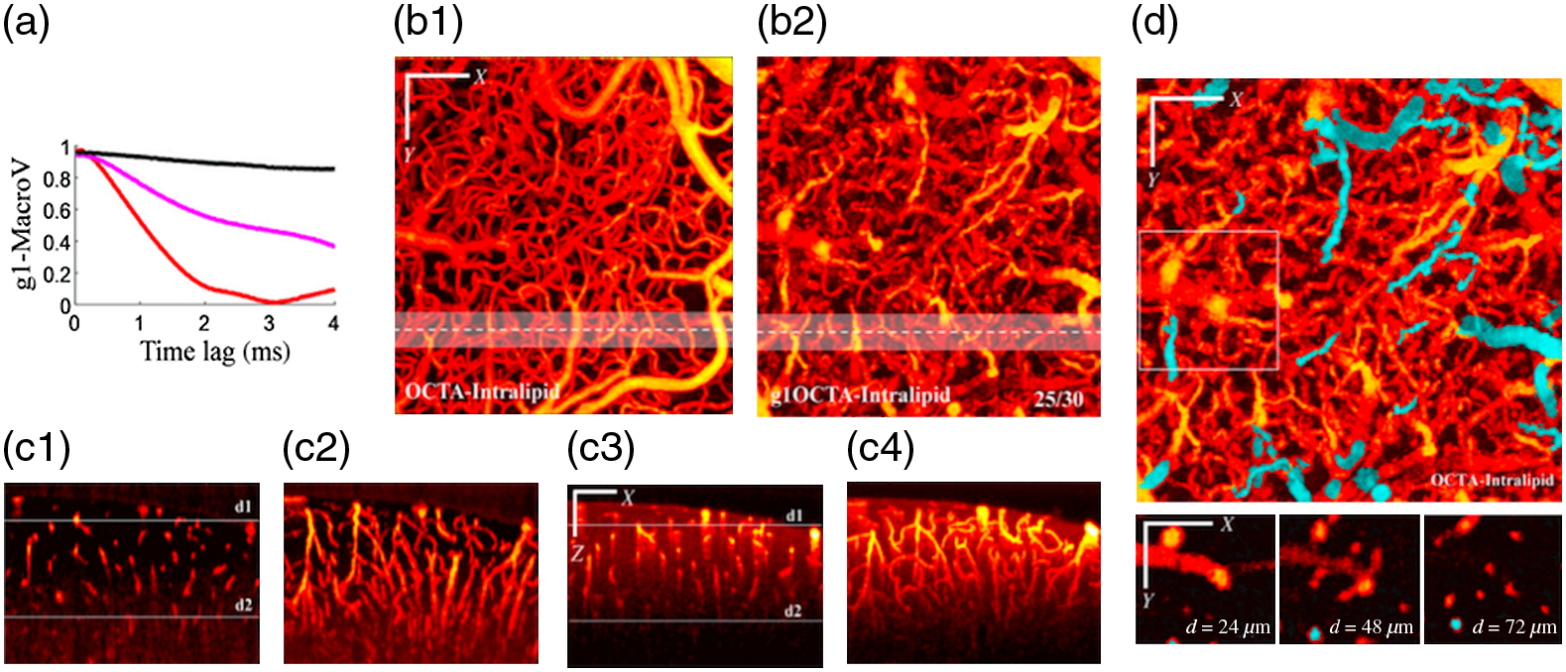
(a) g1(τ) in 4 ms scale showing the decorrelation at different positions. Calculated from Eq. (5). (Black: above the macro-vessel; red, inside the macro-vessel; and magenta, beneath the macro-vessel.) (b1) Regular OCTA *en face* MIP. (b2) g1-OCTA *en face* MIP with intralipid. The value 25/30 represents $n_{\tau }/n_{t}$. (c1) A single B-scan image from white dashed line marked in (b1). (c2) XZ stack (MIP over 50  μm in Y) from white shadow region marked in (b1). (c3) A single B-scan image from white dashed line marked in (b2). (c4) XZ stack (MIP over 50  μm in Y) from white shadow region marked in (b2). (d) g1-OCTA *en face* MIP and *en face* images at depths = 24, 48, and 71  μm showing the ability to identify descending vessels (cyan color). Reproduced with permission, courtesy of Tang et al.

The aforementioned methods are essentially image post-processing approaches. Tang et al.^59^ proposed a repeat A-scan acquisition in combination with a dynamic analysis method (g1-OCTA) to address the blood vessel tail artifacts in OCTA imaging. Compared to the repeat B-scan data acquisition used in traditional OCTA, the repeat A-scan acquisition can realize much shorter data acquisition interval (higher acquisition rate) at the same location, providing the potential to resolve fast dynamics within the vessel lumen and in the tail region. From the decorrelation curves of |g1| shown in Fig. 15(a), one can note that the pixel within the blood vessel (red) has the fastest decorrelation rate, the pixel in the tail region (magenta) has moderate decorrelation rate, and the pixel from the tissue region (black) has merely little decorrelation. It can be also noted that both the pixels within and beneath the blood vessel will have comparable decorrelation if the decorrelation time is long enough. For traditional OCTA acquisition using repeat B-scan acquisition, the time interval (decorrelation time) is usually 4 to 6 ms, therefore, resulting in strong tail artifacts. So, the g1-OCTA employed a shorter decorrelation time that leads to a much smaller decorrelation in the tail region and a similar decorrelation in the vessel region, thus realizing blood vessel tail artifacts suppression. In addition, to address the issue of insufficient decorrelation in small vessels due slow blood flows in the small vessel, intralipid was injected in the circulation to enhance the dynamic contrast. the autocorrelation function $g_{1}(\tau )$ for each voxel was calculated via

$$g_{1}(\tau )=\frac{{\left\langle R^{*}(t)R(t+\tau )\right\rangle }_{t}}{{\left\langle R^{*}(t)R(t)\right\rangle }_{t}},$$ (5)

where R(t) is the complex OCT signal at time t, $R^{*}(t)$ is the complex conjugate, τ is the time lag, and ⟨ ⟩ represents ensemble averaging. Dynamic contrast index $I_{d}$, was calculated for each voxel in this method. $I_{d}$ was defined as the maximum decay of the correlation function since the first time lag as

$$I_{d(x,y,z)}=|g_{1}{\left(1\right)}_{\left(x,y,z\right)}|-\min|g_{1}{\left(\tau \right)}_{\left(x,y,z\right)}|.$$ (6)

Compared to regular OCTA, g1-OCTA realized blood vessel artifacts suppression with enhanced blood flow signals and without broken flows in deep layers that suffered by the subtraction-based methods. In addition, g1-OCTA can also improve the depth-of-field because of noise suppression [in Figs. 15(b) and 15(c)]. Further, the descending vessels could also be identified in the axial direction by analyzing the phase change, as shown in Fig. 15(d). However, compared to the acquisition time of a few seconds for 3D-volume imaging with regular OCTA, g1-OCTA needs 3 to 4 min to acquire data, making it unsuitable for scenarios where long data acquisition is not applicable, such as an eye examine. Nevertheless, g1-OCTA has a good potential in blood flow mechanism study using animal models where long data acquisition time is not a problem.

## 5 Discussion and Conclusion

The blood vessel tail artifacts in an OCTA image are due to the multiple scattering of photons with flowing RBCs. Such elongated light path extends the dynamic signal from vessel lumen to the regions below the lower boundary of blood vessels. In this review, we summarized the existing methods to address the blood vessel tail artifacts issue that prevents OCTA to realize real 3D imaging. The high NA approach suppressed the blood vessel tail artifacts using the short confocal depth to reject multiple scattered photons out of this focus region, but at the expense of repeated data acquisition at multiple focal depth. The subtraction-based methods^15^^,^^53^^,^^54^^,^^57^ remove the tails by subtracting the vessel signal in the superficial layers from the deep layers, which however suffers from broken flows due to the removal of voxels under the large vessels. The PR-OCTA and rbPR-OCTA approaches can alleviate this issue to some extent but still can’t fully address this problem. The Hessian filtering approaches showed good performance in blood vessel tail artifacts suppression and preserve the flow continuity in deep layers, but it relies on high signal-to-background ratio and is vulnerable to false positives. The g1-OCTA approach is based on the dynamic analysis of high rate acquired data (repeat A-scan) and using a shorter decorrelation time to minimize the tail artifacts and preserve the flow continuity in small vessels by employing intralipid to fill the gap between RBCs, but it has the limitation of longer data acquisition time. In addition to the aforementioned methods, the convolutional neural networks (CNN)-based deep learning has also been tested for OCTA data processing. It shows the potential to map microvascular networks with better imaging contrast. However, this method relies heavily on the accuracy of ground truth, which is usually generated by manual labeling, requiring laborious work.^65^^,^^66^ In addition, the deep learning process is usually designated for a specific scenario, such as a specific shape or structure, and it is currently challenging to use deep learning process for complex inputs. However, the blood vasculature is composed of various complex structures. It thus needs to consider all possible scenarios of vessel structures to obtain reliable results. Nevertheless, in combination with deep learning, it is possible to suppress the blood vessel tail artifacts and realize real 3D blood vessel structure imaging with OCTA.

In conclusion, we have reviewed the imaging principle of OCTA, the origin of blood vessel tail artifacts in an OCTA image, and summarized the current methods for blood vessel tail artifacts suppression. The existing techniques have addressed the blood vessel tail artifacts in OCTA to some extent, but none of them have an absolute advantage over the others. Future work may focus on tail artifacts suppression of large vessels while preserving the small vessel flows in the tail artifacts region, thus providing real 3D blood vessel structure imaging with OCTA. In combination with the OCT-based blood flow velocity imaging, it is possible to realize the RBC transit time 3D imaging of the blood vessel network, providing a robust tool for the assessment of this important blood flow parameter.
